# Histologic transformation of lung cancer during pembrolizumab therapy: A case report

**DOI:** 10.1111/1759-7714.13312

**Published:** 2020-01-16

**Authors:** Xiaoyan Si, Yan You, Xiaotong Zhang, Hanping Wang, Mengzhao Wang, Li Zhang

**Affiliations:** ^1^ Division of Pulmonary and Critical Care Medicine Peking Union Medical College Hospital Beijing China; ^2^ Department of Pathology Peking Union Medical College Hospital Beijing China

**Keywords:** Histologic transformation, immune checkpoint inhibitor, non‐small cell lung cancer, pembrolizumab, small cell lung cancer

## Abstract

Immune checkpoint inhibitors that block the programmed death 1/programmed death ligand 1 pathways are widely used to treat advanced lung cancers. There are seldom cases of histologic transformation reported after treatment with immunotherapy. Here, we report the case of a 69‐year‐old man with stage IV lung squamous cell carcinoma. He received pembrolizumab monotherapy and had a partial response. After 22 cycles of pembrolizumab, chest computed tomography (CT) showed a left hilar tumor, bilateral pleural effusion and lymphadenopathy. The cytology of pleural effusion and bronchoscopic biopsy of an intraluminal lesion revealed small cell lung cancer. After two cycles of chemotherapy (etoposide/carboplatin), CT scan revealed shrinkage of lesions. This is the first case of lung squamous cell carcinoma with histologic transformation after treatment with pembrolizumab alone.

## Introduction

**Figure 1:**
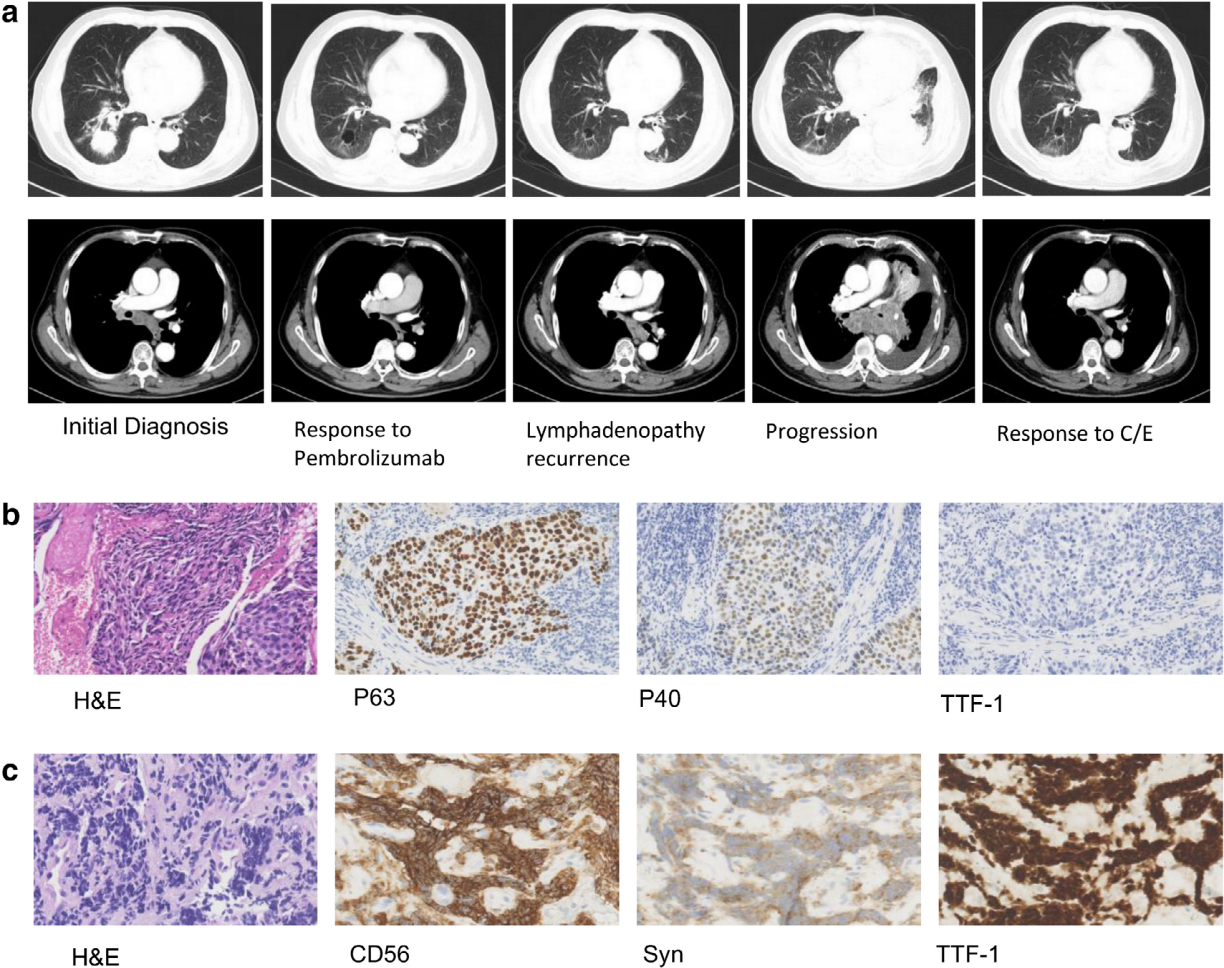
(**a**) On initial diagnosis, chest computed tomography (CT) scan showed a 3.9 cm right lower lobe tumor with right hilar and mediastinal lymphadenopathy, and pericardial effusion. The patient was subsequently treated with pembrolizumab monotherapy and had a partial response. After 22 cycles of pembrolizumab, follow‐up CT scan showed a left hilar tumor, bilateral pleural effusion and lymphadenopathy recurrence. After two cycles of chemotherapy (carboplatin/etoposide/), CT scan revealed shrinkage of the lesions. (**b**) Pathologic findings from the right lower lobe lesion at the time of initial diagnosis showed squamous cell carcinoma (hematoxylin and eosin [H&E] staining, ×200), with positive immunohistochemical staining for P63 and P40 (×200), and negative staining for thyroid transcription factor1 (TTF‐1). (**c**) Pathologic finding from the intraluminal lesion in the left main bronchus at the time of progression showed small cell lung cancer (H&E, ×400), with positive immunohistochemical staining for CD56 (×400), synaptophysin (Syn×400), and TTF‐1(×400).

Immune checkpoint inhibitors (ICIs) that block the programmed death 1 (PD‐1)/programmed death ligand 1 (PD‐L1) pathways, such as pembrolizumab and nivolumab, are widely used to treat advanced lung cancers. They significantly improve overall survival in patients with advanced non‐small cell lung cancer (NSCLC). Histologic transformation has been previously reported in patients with lung cancer who are receiving epidermal growth factor receptor (EGFR) tyrosine kinase inhibitors (TKIs).^1^, ^2^ However, there are seldom cases of histologic transformation reported after treatment with immunotherapy. Here, we present a case of lung squamous cell carcinoma (SCC) with histologic transformation after treatment with pembrolizumab alone (Fig 1).

## Case report

A 69‐year‐old man who had smoked for 48 years presented with a dry cough in October 2017. Chest computed tomography showed a 3.9 cm right lower lobe tumor with right hilar and mediastinal lymphadenopathy, and pericardial effusion. A biopsy from the lower lobe of right lung demonstrated lung SCC, with positive immunohistochemical (IHC) staining for P40, and negative staining for P63 and thyroid transcription factor 1. Lung SCC (cT2aN2M1a, stage IV) was diagnosed. The expression of PD‐L1 was positive (tumor proportion score ≥1%, IHC 22C3 pharmDx assay, Dako North America). The patient then received pembrolizumab 200 mg every three weeks from December 2017. He had a partial response according to Response Evaluation Criteria in Solid Tumors vision 1.1. The mediastinal lymphadenopathy had reappeared on chest computed tomography (CT) scan in January 2019 although the patient had no symptoms. Therefore, treatment with pembrolizumab continued. After receiving 22 cycles of pembrolizumab, he presented with dyspnea in March 2019. Chest CT scan showed a left hilar tumor, bilateral pleural effusion and lymphadenopathy. Serum tumor markers of small cell lung cancer (SCLC) were pro‐gastrin‐releasing peptide (pro‐GRP) 311 pg/mL (0–50), and neuron‐specific enolase (NSE) 155 ng/mL (0–16), while serum tumor markers for SCC were in the normal range. The cell block of pleural effusion revealed SCLC (positive staining for Chromogranin A, CD56, synaptophysin, and thyroid transcription factor 1, and negative for P63). Bronchoscopic biopsy of an intraluminal lesion in the left main bronchus also revealed SCLC (positive staining for Chromogranin A, CD56, synaptophysin, and thyroid transcription factor 1) with negative PD‐L1 expression. Next‐generation sequencing showed a tumor protein 53 (TP53) R342* (the asterix means termination codon) nonsense mutation in the lesion in the left main bronchus. He underwent six cycles of etoposide and carboplatin every three weeks from April 2019. After two cycles of chemotherapy, CT scan revealed shrinkage of lesions and tumor markers had markedly decreased (NSE 12 ng/mL, pro‐GRP 57 pg /mL).

## Discussion

Histologic transformation has been reported as a mechanism of resistance to EGFR‐TKIs and anaplastic lymphoma kinase TKIs. Mechanisms for histologic transformation may be related to pluripotent cancer stem cell differentiation and tumor heterogeneity.

We reviewed previous case reports of histologic transformation where resistance to ICIs had occurred (Table 1). However, most of the cases reported had other treatment besides ICIs before resistance occurred. Therefore, effects from previous radiation and chemotherapy on histologic transformation must be considered. To the best of our knowledge, this is the first case reporting transformation from SCC to SCLC due to treatment with pembrolizumab alone.

**Table 1:** Case reports of histologic transformation treated with ICIs

Case	Sex/age	Initial histology	Treatment	Transformed histology	Treatment after transformation	Efficacy of carboplatin/ etoposide
Hsu *et al*. 3	M/69	ADC, EGFR‐, ALK‐	Docetaxel; vinorelbine; radiotherapy; pembrolizumab	SCC with sarcomatoid changes, PD‐L1+	pembrolizumab	
Imakita, *et al*.^4^	M/75	Poorly differentiated NSCLC, EGFR‐, ALK‐	Docetaxel/ bevacizumab; nivolumab	SCLC, PD‐L1‐	amrubicin	PD
Nagasaka *et al*.^5^	M/71	SCC	Radiotherapy; carboplatin/ paclitaxel; nivolumab	ADC		
Abdallah, *et al*.^6^	M/65	ADC, EGFR‐, ALK‐	Carboplatin/ pemetrexed; nivolumab	SCLC	Carboplatin/ etoposide	PR
	M/68	Moderately differentiated SCC	Surgery; carboplatin/ paclitaxel/ pembrolizumab	SCLC	Carboplatin/ etoposide; radiotherapy	CR
		Poorly differentiated NSCLC				
Iams, *et al*.^7^	F/67	Poorly differentiated ADC, KRAS+	Carboplatin/ paclitaxel; nivolumab	SCLC, KRAS+, TP53+, RB+	Carboplatin/ etoposide; paclitaxel	PR
	F/75	ADC, KRAS+	nivolumab	SCLC, KRAS+, TP53+	Carboplatin/ etoposide; nivolumab/ ipilimumab; irinotecan	SD
Okeya, *et al*.^8^	M/66	ADC, ALK‐, PD‐L1+	Carboplatin/ paclitaxel/ bevacizumab; pembrolizumab	SCLC	Carboplatin/ etoposide; amrubicin	PR

ADC, adenocarcinoma; ALK, anaplastic lymphoma kinase rearrangement; EGFR, epidermal growth factor receptor mutation; ICIs, immune checkpoint inhibitors; NSCLC, non‐small cell lung cancer; PD, progressive disease; PD‐L1, programmed death ligand 1; PR, partial response; RB1, retinoblastoma 1 mutation; SCC, squamous cell carcinoma; SCLC, small cell lung cancer; SD, stable disease; TP53, tumor protein 53 mutation.

In our case, the initial pathological findings showed SCC. Both the right lower lobe lesion and lymphadenopathy responded to pembrolizumab. After progression, pathological findings from pleural effusion and intraluminal lesion, elevated serum tumor markers and good response to carboplatin/etoposide were concordant with SCLC. Although a mixed type of tumor should be considered due to limited specimen, the clinical and pathological characteristics supported histologic transformation.

The TP53 mutation was detected after histologic transformation. Although the TP53 status at initial diagnosis was unknown, the presence TP53 mutation may increase the risk of SCLC transformation.^7^, ^9^ The expression of PD‐L1 changed after histologic transformation in our case. Hsu *et al*.^3^ reported the PD‐L1 expression disappeared after discontinuation of pembrolizumab. It is meaningful to explore dynamic PD‐L1 expression. Serum tumor markers of SCLC were also elevated in our case and other cases reporting SCLC transformation after treatment with ICIs.^4^, ^8^

Most patients with transformed SCLC respond to carboplatin and etoposide, with overall survival of approximately five to 18 months.

There were some limitations in our case report. First, genetic status of SCC was not evaluated due to an inadequate specimen. Recent studies have revealed that transformation to SCLC is associated with inactivation of RB1 and TP53.^9^ Therefore, genetic status will help us understand how resistance and histologic transformation proceed. Second, there was the possibility of a second primary cancer. However, we believe our case presented NSCLC transformation to SCLC transformation because of recurrence occurring from mediastinal lymph nodes, which was also involved in SCC. Third, the possibility of a combined SCLC with SCC could not be ruled out due to a needle biopsy at the initial diagnosis.

The possibility of tumor transformation should be considered during treatiment with ICIs. It is also important to monitor serum tumor markers of SCLC during treatment with ICIs and perform a repeat biopsy when resistance to ICIs occurrs. Chemotherapy with carboplatin and etoposide is a treatment choice when a transformed SCLC occurs.

## Disclosure

All authors declare that they have no conflicts of interest.
